# Differential Requirement for Pten Lipid and Protein Phosphatase Activity during Zebrafish Embryonic Development

**DOI:** 10.1371/journal.pone.0148508

**Published:** 2016-02-05

**Authors:** Miriam Stumpf, Jeroen den Hertog

**Affiliations:** 1 Hubrecht Institute, Koninklijke Nederlandse Akademie van Wetenschappen (KNAW) and University Medical Center Utrecht, Utrecht, the Netherlands; 2 Institute of Biology Leiden, Leiden University, Leiden, the Netherlands; Florida International University, UNITED STATES

## Abstract

The lipid- and protein phosphatase PTEN is one of the most frequently mutated tumor suppressor genes in human cancers and many mutations found in tumor samples directly affect PTEN phosphatase activity. In order to understand the functional consequences of these mutations *in vivo*, the aim of our study was to dissect the role of Pten phosphatase activities during zebrafish embryonic development. As in other model organisms, zebrafish mutants lacking functional Pten are embryonically lethal. Zebrafish have two *pten* genes and *pten* double homozygous zebrafish embryos develop a severe pleiotropic phenotype around 4 days post fertilization, which can be largely rescued by re-introduction of *pten* mRNA at the one-cell stage. We used this assay to characterize the rescue-capacity of Pten and variants with mutations that disrupt lipid, protein or both phosphatase activities. The pleiotropic phenotype at 4dpf could only be rescued by wild type Pten, indicating that both phosphatase activities are required for normal zebrafish embryonic development. An earlier aspect of the phenotype, hyperbranching of intersegmental vessels, however, was rescued by Pten that retained lipid phosphatase activity, independent of protein phosphatase activity. Lipid phosphatase activity was also required for moderating pAkt levels at 4 dpf. We propose that the role of Pten during angiogenesis mainly consists of suppressing PI3K signaling via its lipid phosphatase activity, whereas the complex process of embryonic development requires lipid and protein phosphatase of Pten.

## Introduction

*PTEN* (Phosphatase and tensin homolog) is one of the most frequently mutated tumor suppressor genes in spontaneous cancers [1, 2] and germline mutations of *PTEN* have been associated with PTEN hamartoma tumor syndromes (PHTS), such as Cowden syndrome and Bannayan-Riley-Ruvalcaba syndrome [3–5], and with autism-spectrum disorders [6, 7]. Moreover, homozygous loss of germline *PTEN* is incompatible with life in higher eukaryotes [8–12], illustrating its essential functions for multicellular organisms since early embryonic development.

A prominent function of the tumor suppressor PTEN is its lipid phosphatase activity towards phosphatidylinositol (3, 4, 5)-trisphosphate (PIP3), which makes PTEN the main antagonist of the cell proliferation and cell survival promoting phosphatidylinositol-3-kinase (PI3K)/ Akt (also known as protein kinase B, PKB) signaling pathway [13]. Little-known, in contrast, is its dual-specificity protein phosphatase activity against phosphotyrosine (pTyr), phosphoserine (pSer) and phosphothreonine (pThr), which was discovered and characterized [14] shortly after the identification of *PTEN* as one of the most frequently mutated tumor suppressor genes in human cancers. Nonetheless, the main attention so far has been drawn to the lipid phosphatase activity of PTEN, partly due to the early identification of germ line mutations in *PTEN* in Cowden syndrome patients, that are particularly affecting the protein’s lipid phosphatase activity [3, 15].

As in other members of the PTP (protein tyrosine phosphatase) family, the catalytic core of the PTEN PTP domain contains a cysteine, in this case Cys124, which is essential for dephosphorylation of substrates [16, 17]. Mutation of PTEN Cys124 to serine, C124S, which has been associated with spontaneous cancer [18], completely abolishes PTEN phosphatase activity towards inositol phospholipids or phosphorylated proteins [19]. Another point mutation within the catalytic core, G129E, which has been first identified in Cowden syndrome patients, only affects lipid- but not protein phosphatase activity of PTEN [3, 20]. In tumor specimens, Gly129 has been found mutated to either glutamate, G129E; arginine, G129R [14] or valine, G129V (http://cancer.sanger.ac.uk/cosmic). Due to its specific loss of lipid phosphatase activity but not protein phosphatase activity, PTEN G129E has to date been a valuable tool to study the contribution of each of the two enzymatic activities of PTEN *in vitro* [14, 20–25] and *in vivo* [26]. Upon the discovery of the PTEN related phosphatase TPIP (Transmembrane phosphoinositide 3-phosphatase and tensin homolog) [27], which lacks protein phosphatase activity, and based on the homologous region shared with PTEN, Leslie et al. generated a PTEN mutant, Y138L, which conserves PTEN lipid phosphatase activity but lacks PTEN protein phosphatase activity [28]. Mutation of Tyr138 to cysteine, Y138C, has further been identified in a small cell lung carcinoma cell line, indicating that, though apparently with less frequency, also lack of PTEN protein phosphatase activity is positively selected for in some types of cancer [29].

These three phosphatase mutants of PTEN, C124S, G129E and Y138L, have been used in different studies, *in vitro* and *in vivo*, to functionally dissect PTENs distinct phosphatase activities [26, 28, 30, 31]. However, rescue experiments in living organisms using all three phosphatase mutants of PTEN, have not been done systematically yet. In cell based assays, the requirement for lipid phosphatase activity of PTEN to suppress cell proliferation and colony formation was confirmed definitively [22, 32, 33], whereas the importance of the protein phosphatase activity in this process is debatable and depends on the kind of assay performed [28, 29, 34]. However, the PTEN protein phosphatase activity seems to be required for glial cell migration [35–37], for regulating differentiation of neuronal progenitor cells [31], for suppression of cell invasion *in vitro* (Matrigel assay) and for suppression of endothelial to mesenchymal transition (EMT) *in vivo* [28, 29, 38]. The identity of the protein substrates of PTEN remains to be determined definitively. Some of the protein phosphatase-dependent functions of PTEN have recently been attributed to PTEN autodephosphorylation rather than to dephosphorylation of other target proteins [29, 37, 39–41].

The role of PTEN lipid and protein phosphatase activity during embryonic development has not been studied in much detail yet, since it requires the availability of a suitable animal model. Analysis of mouse *PTEN* knockouts and knockins during embryonic development is hampered by embryonic lethality of PTEN knockouts at day E8.5 [8] and because development takes place *in utero*. We use zebrafish to study protein function during development. Zebrafish is a great model, because of extra-uterine development, the number of embryos generated (∼200 per female, weekly), the transparency of the egg and the variety of techniques to (re-) introduce genetic information (synthetic RNA, DNA) at the one-cell stage.

Zebrafish have two *pten* genes, *ptena* and *ptenb*, that have redundant functions [9, 10]. We generated *ptena-/-* and *ptenb-/-* fish lines by target-selected gene inactivation (TSGI) and inbred them to obtain *ptena+/-ptenb-/- and ptena-/-ptenb+/-* fish lines that we use for our functional rescue experiments [9, 42, 43]. While their heterozygous and single homozygous siblings are viable and fertile, double homozygous embryos that lack all Pten activity develop a pleiotropic phenotype, characterized by massive heart edema, craniofacial defects, aberrant pigmentation and shorter body axis. Those embryos die around 5 days post fertilization (dpf). We previously characterized the pleiotropic phenotype of double homozygous *ptena-/-ptenb-/-* embryos at different developmental stages [9], unveiled the formation of hemangiosarcomas in adult *pten* haploinsufficient zebrafish [44] and further studied the role of Pten in zebrafish angiogenesis [43] and hematopoiesis [45].

Angiogenesis, the formation of new blood vessels from the existing vasculature, is a common hallmark of solid tumor progression and the underlying signaling pathways, especially VEGFR-signaling, have been extensively studied as therapeutic targets. We recently described that *pten* double homozygous embryos have constitutively elevated pAkt and vegfaa levels [9, 43, 44], accompanied by enhanced angiogenesis that can be visualized from 3dpf onwards using confocal microscopy in *ptena-/-ptenb-/-* zebrafish embryos in the transgenic Tg(*kdrl*:*eGFP*) background [43]. This vasculature hyperbranching phenotype is rescued by treatment with the PI3K-specific inhibitor LY294002 but also by micro-injection of synthetic *pten* mRNA [43].

In this study we investigated which Pten phosphatase activity, lipid or protein phosphatase, is required for normal zebrafish embryonic development and angiogenesis. We tested the capacity of phosphatase activity mutants of Pten to rescue the pleiotropic phenotype at 4dpf when injected as synthetic mRNA at the one-cell stage. Wild type Pten, but none of the phosphatase mutants, was able to rescue the pleiotropic phenotype at 4dpf. In contrast to the pleiotropic phenotype at 4dpf, the hyperbranching vessel phenotype at 3dpf was rescued by both lipid phosphatase active Pten constructs, Pten wild type and Pten Y138L. Further, lipid phosphatase active Pten decreased activated Akt (pAkt) levels in *pten* double homozygous zebrafish embryos at 4dpf. We conclude that Pten lipid phosphatase activity is required to regulate correct vessel formation and pAkt levels during zebrafish embryogenesis, whereas the Pten protein phosphatase activity seems dispensable for regulating angiogenensis. For correct zebrafish embryonic development, however, both lipid and protein phosphatase activities appear to be required.

## Material and Methods

### Ethics statement

All procedures involving experimental animals described in this manuscript were approved by the local animal experiments committee (Koninklijke Nederlandse Akademie van Wetenschappen-Dierexperimenten commissie protocol HL05.1501) and performed according to local guidelines and policies in compliance with national and European law.

### Fish line

Zebrafish were maintained and the embryos were staged as previously described [46]. The lines Tg(*kdrl*:*eGFP*) and *ptena-/-*, *as well as ptenb* -/- were previously described [9, 47, 48].

### Constructs, mRNA synthesis and Micro-injections

The Ptenb-mCherry and Ptenb-eGFP construct were obtained by amplification of *ptenb* from the vectors described in [9]. The PCR-product and the pCS2+mCherry or pCS2+eGFP vectors were digested with BglII and *ptenb* was subsequently ligated into pCS2+mCherry and pCS2+eGFP. The point mutations in the PTP domain were introduced by site-directed mutagenesis. The constructs were linearized with NotI and to synthetize 5’ capped sense RNA, the mMessage mMachine SP6 kit (Ambion) was used. mRNA injections were performed at the one-cell stage as described using a total of 300 pg of mRNA.

### Lysis

Zebrafish embryos at 4dpf were anesthetized with 16mg/ml 3-amino benzoic acid ethylesther (MS-222) and cut in half (just below the yolk sack extension). The tail was lysed for genotyping and the head/trunk region was subjected to a protein lysis protocol for subsequent immunoblotting (see below).

### Immunoblotting

Zebrafish embryos were lysed at 4dpf in 25mM HEPES (pH7,4), 125mM NaCl, 0,25% Deoxycholate, 10mM MgCl_2_, 1mM EDTA, 1% Triton X-100, 10% Glycerol buffer containing proteinase and phosphatase inhibitors. Samples were lysed for 30min and subsequently subjected to sonication with the Bioruptor (settings: high intensity, 15min, 1min on/off cycles). Samples were run on a 10% SDS-PAGE gel, transferred to a PVDF membrane and stained with Coomassie Blue to verify equal loading. The blots were probed with antibodies specific to pAkt (1:2000), Akt (1:1000) and PTEN (1:1000); (all Cell Signaling) and Tubulin (1:3000); (Calbiochem). For signal detection, enhanced chemiluminescence (Thermo Scientific kit) was diluted 1:2 in home- made ECL.

### Confocal microscopy and Analysis

Zebrafish embryos were anesthetized at 3dpf and laterally mounted on glass bottom dishes (Greiner bio one) in 0,5% agarose (type V, Sigma Aldrich) in E3 embryo medium containing 16mg/ml 3-amino benzoic acid ethylesther (MS-222) to block contractile movements. Confocal microscopy was performed using a Leica TCS SPE, 20x objective. Z-stacks (step size 2μm) of the vasculature in the tail region, right below the yolk sack extension, were acquired for every embryo at 28.5°C. Image J software (http://rsb.info.nih.gov./ij/) was used to generate z-projection images of the zebrafish embryonic vasculature.

### Statistics

Significance of the frequency of occurrence of the “hyperbranching” vasculature phenotype in the different experimental conditions compared to the non-injected control was assessed using two-tailed Fisher’s exact test. Results were considered significant when p<0.05 (p values * p<0.05, ** p<0.01, *** p<0.001).

## Results

### Pten phosphatase mutants

**Fig 1:**
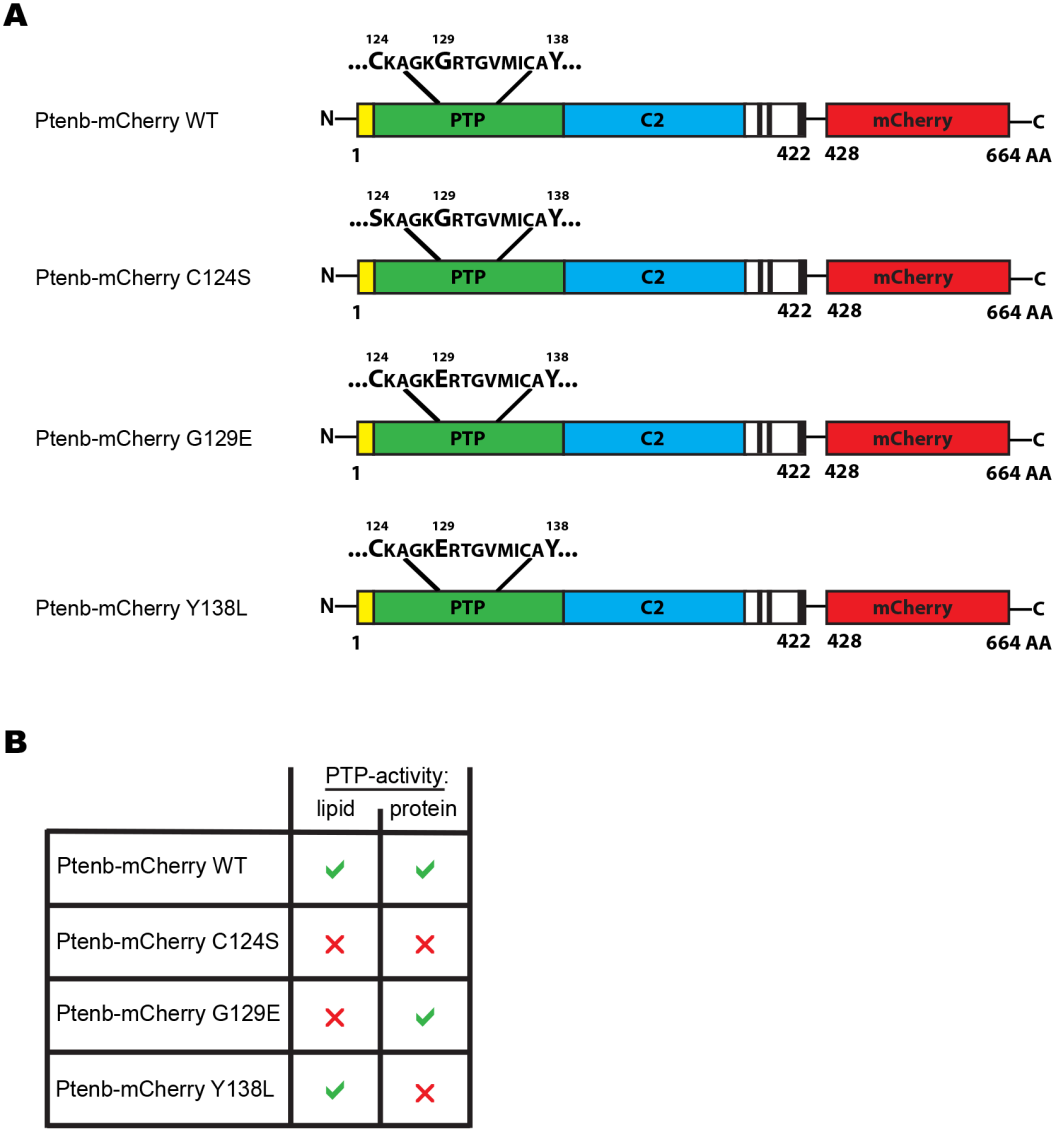
Schematic representation of the Ptenb-mCherry WT, C124S, G129E and Y138L constructs and their enzymatic activities. **(A)** Wild type Ptenb (long splicing variant, 422 amino acids) consists of the N-Terminus (yellow), the PTP-domain (green), the C2 domain (blue) and the C-terminus (black and white). The red fluorescent protein mCherry (red) is C-terminally tagged in frame to all Ptenb constructs. The point mutations of interest were introduced into the PTP-domain by site-directed mutagenesis. **(B)** Phosphatase activities of the Ptenb mutants: Wild type Ptenb posseses phosphatase activity against phospholipids and phosphorylated peptide sequences, which can both be completely abrogated by mutation of the catalytic cysteine, Cys124 to serine (C124S). Mutation of Gly129 to glutamate (G129E) results in loss of Ptenb lipid phosphatase activity while protein phosphatase activity is retained. Mutation of Tyr138 to leucine (Y138L), renders a protein-phosphatase-dead Ptenb that retains lipid phosphatase activity.

In order to study the role of Pten phosphatase activities during zebrafish embryonic development, we introduced point mutations, that are known to disrupt—fully or partially—the enzymatic activity of human PTEN, in the PTP domain of the zebrafish homologue, Ptenb (Fig 1A). The sequence conservation of the catalytic domain between human PTEN and zebrafish Pten is extremely high. All residues that contact the substrate, including Asp92, His93, Lys125, Ala126, Gly127, Lys128, Gly129, Arg130, Thr167, Ile168 and Gln171, and particularly the catalytic site cysteine of PTEN, Cys124, [49] are conserved in Ptenb. Therefore, we believe that mutation of C124S, G129E or Y138L in Ptenb affects the catalytic activities of these mutants in a similar manner as in human PTEN. We previously reported that zebrafish, as many other teleosts, have two *pten* genes, *ptena* and *ptenb*, that have redundant functions during embryonic development [9] and that both *ptena* and *ptenb* largely rescued the morphological defects in *ptena-/-ptenb-/-* zebrafish [43]. For our approach, we used *ptenb* and mutants. We first generated constructs encoding fluorescent protein-tagged Ptenb, using either eGFP or mCherry, which allowed us to check for correct microinjection and protein expression. Subsequently we created the phosphatase mutants of Ptenb-mCherry and Ptenb-eGFP (not depicted in the schematic) by site-directed mutagenesis (Fig 1A). Mutation of the catalytic cysteine, Cys124, to serine abolishes all phosphatase activity, whereas mutation of Gly129 to glutamate renders a lipid phosphatase inactive Ptenb that retains protein phosphatase activity. Mutation of Tyr138 to leucine on the contrary abolishes protein phosphatase activity, but not lipid phosphatase activity (Fig 1B).

### Both Pten phosphatase activities are required to rescue the *ptena-/-ptenb-/-* pleiotropic phenotype

**Fig 2:**
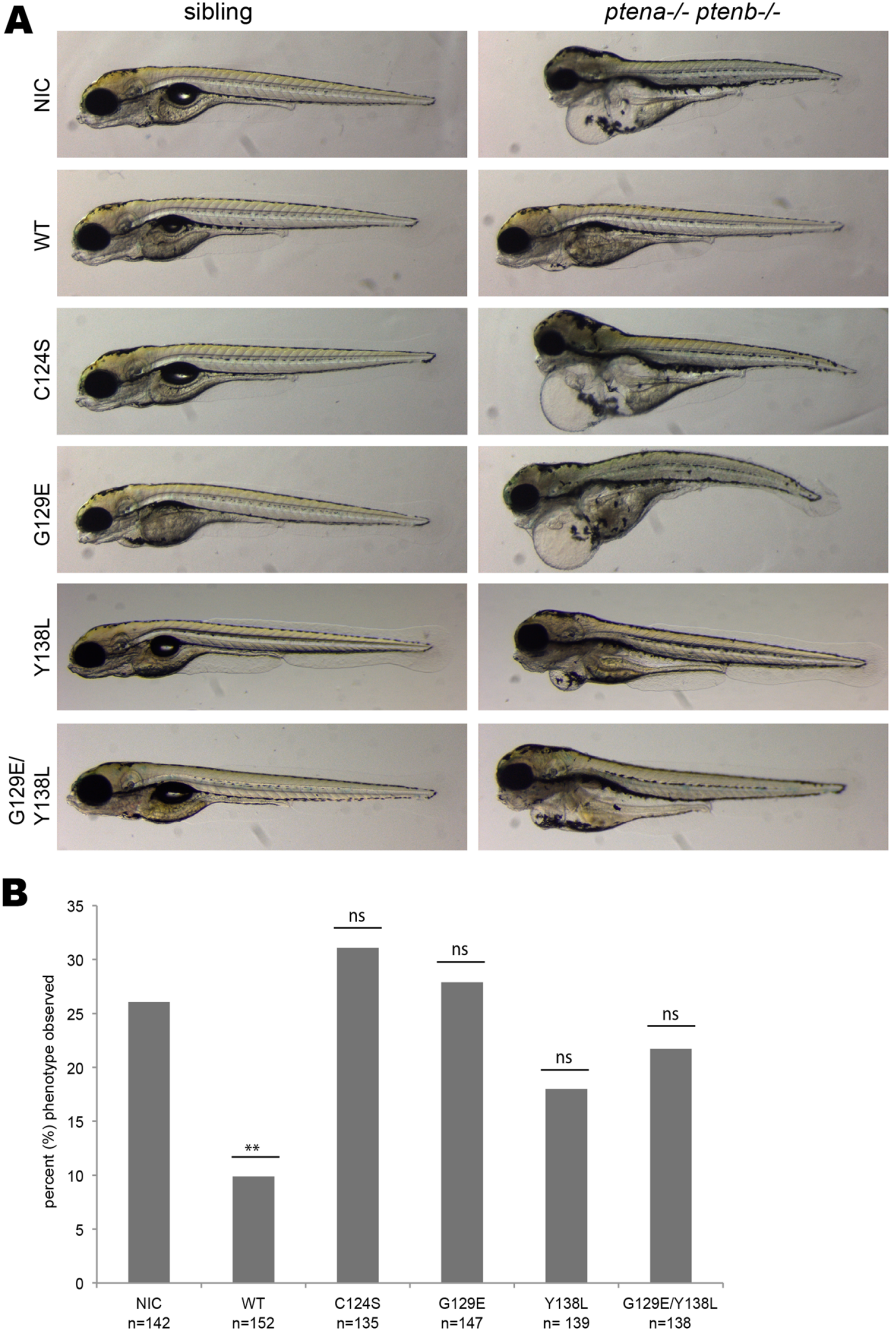
Both Pten phosphatase activities are required to rescue the *ptena-/-ptenb-/-* pleiotropic phenotype. Zebrafish embryos from a *ptena+/- ptenb-/-* incross were injected with either wild type Ptenb-mCherry, Ptenb-mCherry C124S, Ptenb-mCherry G129E or Ptenb-mCherry Y138L encoding synthetic mRNA at the one-cell stage. **(A)** At 4 dpf the embryos were submitted to brightfield microscopy and analyzed for the pleiotropic phenotype. Pictures show representative, genotyped embryos. Non-injected control embryos (NIC) were included for reference. **(B)** Quantification of the embryos showing the typical *ptena*-/-*ptenb*-/- pleiptropic phenotype at 4dpf. In the non-injected control (NIC), approximately 25% of the embryos showed the characteristic phenotype (Mendelian segregation). Only wild type Ptenb-mCherry rescued the pleiotropic *ptena*-/-*ptenb*-/- phenotype significantly at 4dpf. The statistical significance of each of the conditions compared to the non-injected control was determined using two-tailed Fisher’s exact test and is indicated in the bar graph (ns = not significant, * = p-value < 0,05, ** = p-value < 0,01, *** = p-value < 0,001).

For our functional rescue assay, we microinjected embryos of a *ptena+*/-*ptenb-/-* zebrafish incross at the one-cell stage with 300pg of synthetic Ptenb-mCherry WT or phosphatase mutant mRNA. At 4dpf, we performed brightfield microscopy of the whole embryos to assess their overall phenotype. Double homozygous *ptena-/-ptenb-/-* embryos displayed severe developmental defects, including massive heart and abdominal edemas, craniofacial defects, aberrant pigmentation and reduced body axis extension (Fig 2A, non-injected control (NIC)). These defects became visible at 4dpf and were likely provoked by aberrant cell proliferation and enhanced cell survival [9]. Micro-injection of 300 pg synthetic *ptenb* mRNA largely rescued the pleiotropic defects of *ptena-/-ptenb-/-* embryos, whereas injection of 300 pg *ptenb* mRNA did not induce any phenotypes in siblings (Fig 2A, WT). Although most features of the pleiotropic phenotype were rescued by re-introduction of wild type Ptenb, a complete rescue was not achieved. This is probably due to a decrease in mRNA levels over time and to a certain degree of mosaicism that may occur during the early cleavage stages. For quantification (Fig 2B), we therefore considered a highly improved phenotype, like the one of the Ptenb-mCherry WT-injected embryo depicted in Fig 2A, as “rescued”. In this assay, we used the phosphatase mutants of Ptenb in parallel to WT Ptenb in order to assess their capacity to rescue the *ptena-/-ptenb-/-* phenotype. Both lipid-phosphatase inactive mutants, Ptenb-mCherry C124S and Ptenb-mCherry G129E, were not capable of rescuing the pleiotropic phenotype at all. The *pten* double homozygous embryos microinjected with these two mutants closely resembled the non-injected double homozygous siblings (Fig 2A). Micro-injection of Ptenb-mCherry Y138L seemed to considerably alleviate the severity of the phenotype but did not fully rescue it. Yet, compared to the non-injected controls, Y138L injection is a great amelioration. Ptenb-eGFP G129E and Ptenb-mCherry Y138L RNA were co-injected each at half of the dose of the single injections to avoid inadvertant effects of microinjection of too much synthetic mRNA. However, these co-injections did not rescue the *pten* double homozygous phenotype either. In fact, the phenotype appeared much more severe than the one of the Ptenb-mCherry Y138L injected embryos but not as severe as the non-injected control.

According to Mendelian law, one quarter of the offspring of a *ptena+/-ptenb-/-* incross, 25%, is expected to be double homozygous. Counting the number of embryos displaying the *pten* double homozygous phenotype, compared to the total number of embryos in each experimental condition allowed us to assess the rescue capacity of each Ptenb-mCherry construct and to perform statistical analysis (Fig 2B). As expected, the occurrence of the *pten* double homozygous phenotype in the non-injected control was about 25%, which was significantly reduced, to about 10%, by micro- injection of Ptenb-mCherry WT. Ptenb-C124S and Ptenb-G129E did not rescue the morphological defects in *ptena-/-ptenb-/-* embryos. Although there appeared to be a positive effect of Ptenb-mCherry Y138L expression on the number of phenotypes observed, this was not statistically significant and neither was co-expression of Ptenb-G129E and Ptenb-Y138L. We conclude that for normal embryonic development in zebrafish, both phosphatase activities of Pten are required, because none of the Ptenb phosphatase mutants rescued the morphological defects in *ptena-/-ptenb-/-* mutants to the same extent as wild type Ptenb.

### Pten lipid phosphatase activity is sufficient to rescue the hyperbranching vasculature phenotype of *ptena-/-ptenb-/-* zebrafish embryos

**Fig 3:**
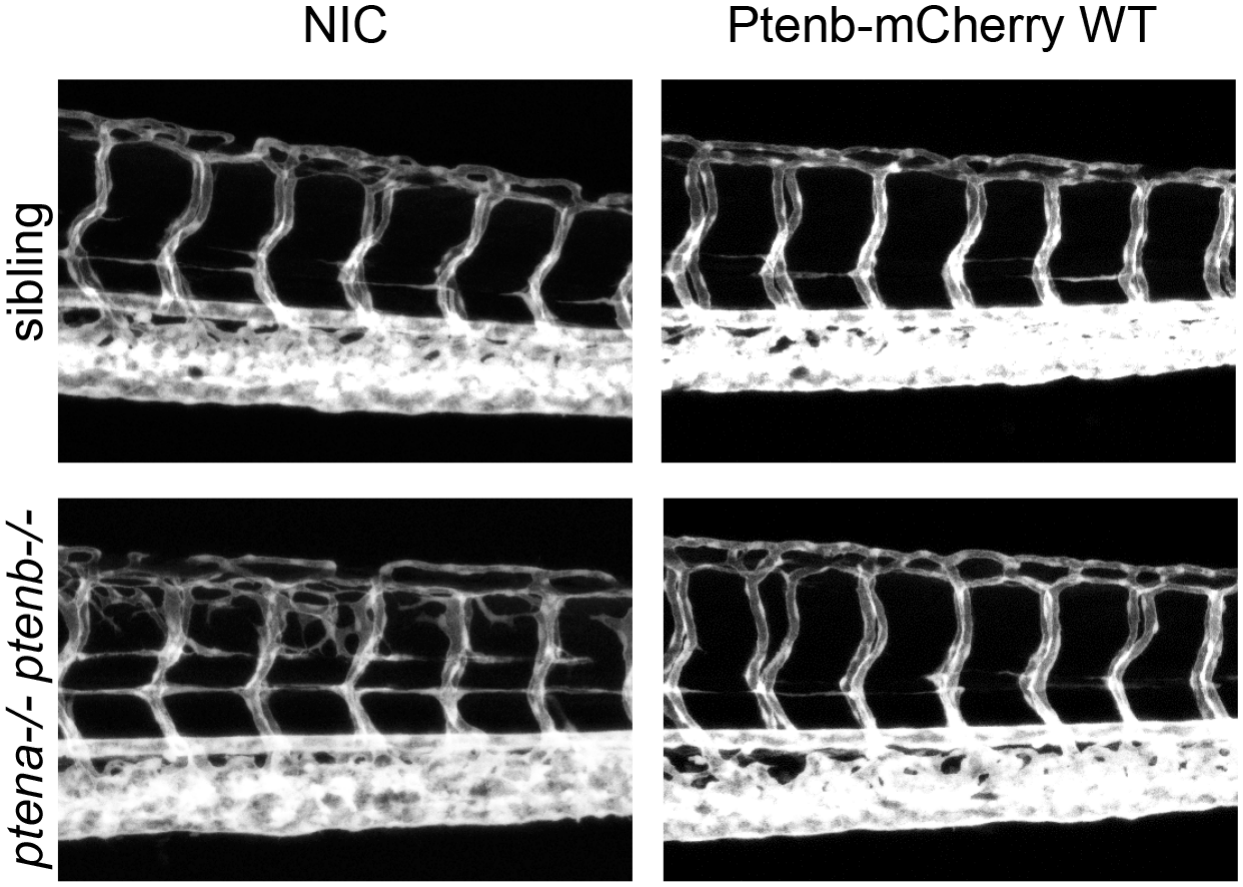
The hyperbranching vasculature phenotype, observed in Pten double homozygous zebrafish embryos at 3dpf, can be rescued by wild type Pten. Zebrafish embryos from a Tg(*kdrl*:*eGFP) ptena+/-ptenb-/-* incross were microinjected at the one-cell stage with 300 pg synthetic mRNA encoding Ptenb-mCherry. At 3dpf the embryos were analyzed for the hyperbranching vessel phenotype by confocal live imaging on a Leica TCS-SPE microscope (anterior to the left, 20x objective, 2μm z-stacks). Subsequently, the embryos were genotyped. Pictures show the trunk region distal from the urogenital opening of representative embryos; non-injected control embryos (control) were included for reference.

**Fig 4:**
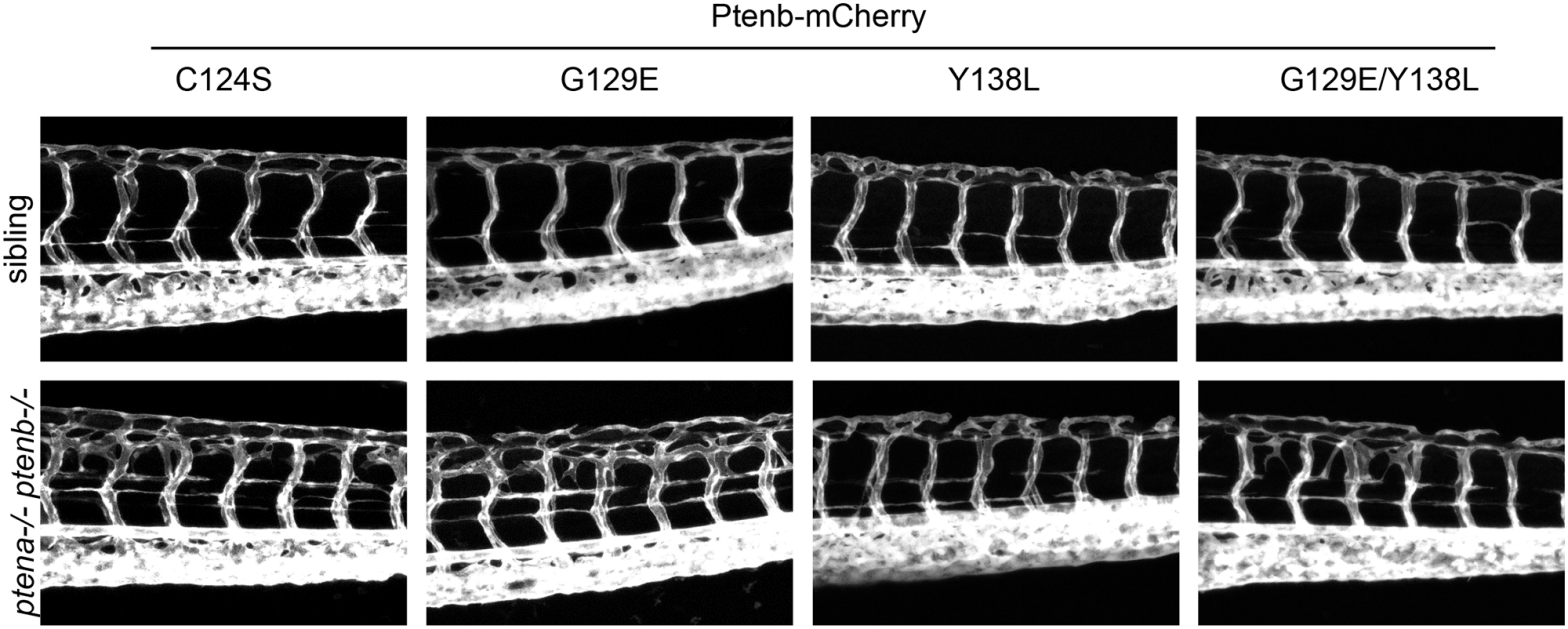
Pten lipid phosphatase activity is required to rescue the hyperbranching vasculature phenotype observed in Pten double homozygous zebrafish embryos at 3dpf. Zebrafish embryos from a Tg(*kdrl*:*eGFP) ptena+/-ptenb-/-* incross were microinjected at the one-cell stage with 300 pg synthetic mRNA encoding Ptenb-mCherry WT (not shown here, see Fig 3) or with either Ptenb-mcherry C124S, Ptenb-mcherry Y138L, Ptenb-mCherry G129E or with 150 pg of each, Ptenb-eGFP G129E and Ptenb-mCherry Y138L. At 3dpf, the embryos were analyzed for the hyperbranching vessel phenotype by confocal live imaging on a Leica TCS-SPE microscope (anterior to the left, 20x objective, 2μm z-stacks). Pictures show the trunk region distal from the urogenital opening of representative genotyped embryos. Both Ptenb WT (shown in Fig 3) and Ptenb Y138L rescue the hyperbranching phenotype observed at 3dpf.

**Fig 5:**
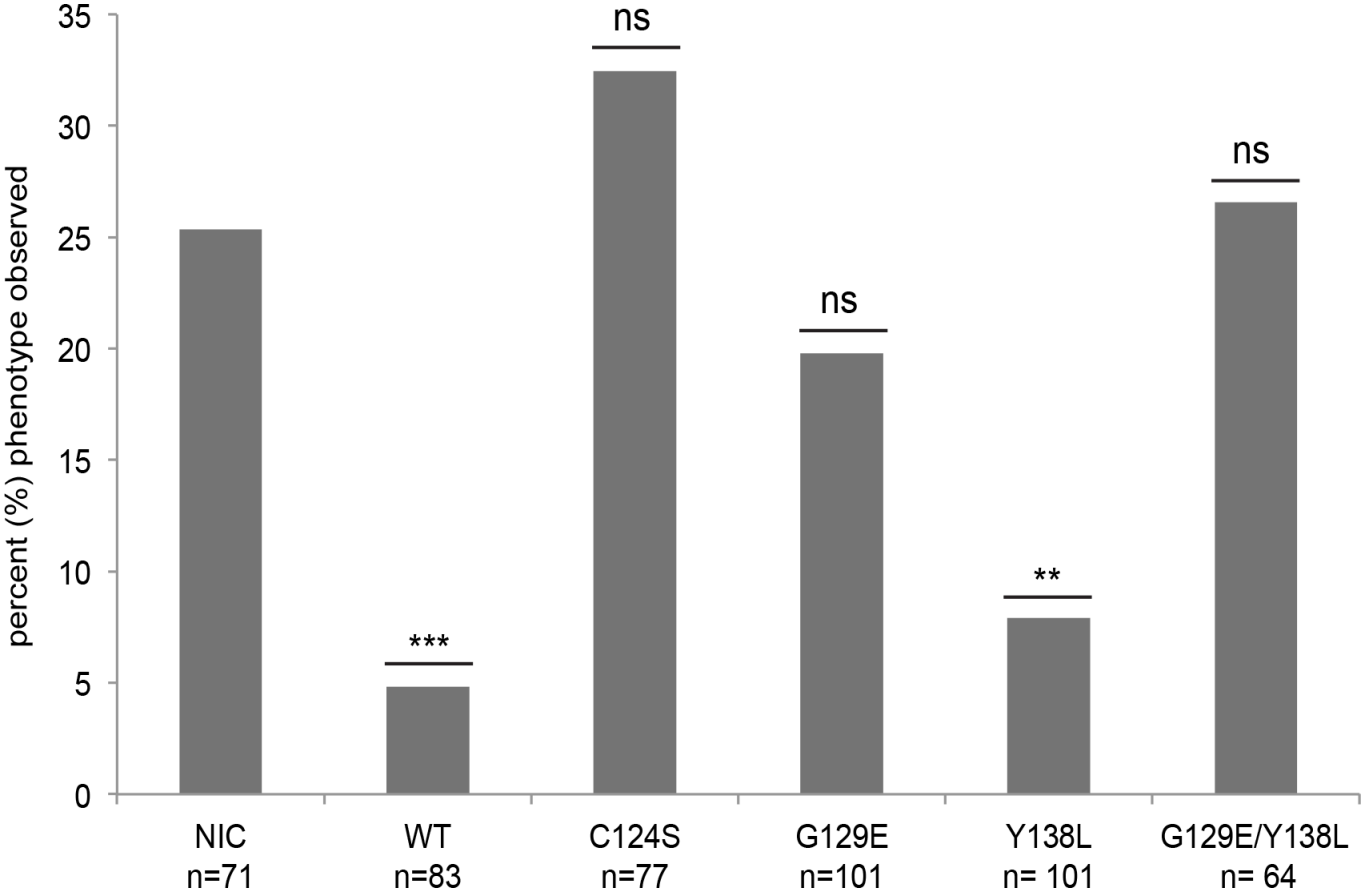
Only lipid phosphatase-active Pten rescues the hyperbranching vasculature phenotype at 3dpf. Quantification of the number of embryos showing the typical *ptena*-/-*ptenb*-/- hyperbranching intersegmental vessel phenotype at 3dpf. Both Ptenb-mCherry WT and Ptenb-mcherry Y138L rescue the characteristic *ptena*-/-*ptenb*-/- hyperbranching phenotype while the other phosphatase mutants do not. The statistical significance was determined using two-tailed Fisher’s Exact test. (ns = not significant, * = p-value < 0,05, ** = p-value < 0,01, *** = p-value < 0,001).

Morphological defects in *ptena-/-ptenb-/-* mutants develop relatively late during embryonic development, at 4dpf. The mCherry signal is already hard to detect at this stage, indicating that a big proportion of the injected mRNA might already have been degraded. Therefore, we decided to focus on a feature of the phenotype that emerges earlier during development: the hyperbranching of the vasculature at 3dpf, which we imaged using a *kdrl*:*eGFP* transgenic zebrafish line [43]. The characteristic trait of this phenotype is the correct development of the intersegmental vessels (ISV), which subsequently produce excessive sprouting, starting at around 70–72 hpf, and quickly evolve into a branch-like meshwork that intrudes into the somite tissue (Fig 3; NIC). Like the pleiotropic phenotype at 4dpf, hyperbranching of the vasculature was largely rescued by re-introduction of Pten [43] (Fig 3). We micro—injected Ptenb WT, Ptenb C124S, Ptenb G129E and Ptenb Y138L or a combination of Ptenb G129E and Y138L RNA in zebrafish eggs of a *ptena+/-ptenb-/-* incross with transgenic Tg(*kdrl*:*eGFP*) background, at the one-cell stage. At 3 dpf, the embryos were subjected to confocal *in vivo* imaging of the vasculature (Figs 3 and 4). The Z-projections of the stacks were analyzed for the occurrence of the hyperbranching phenotype and accordingly categorized into either “normal” or “hyperbranching” phenotype for quantification and statistical analysis (Fig 5). Representative images of embryos of this experiment at 3dpf are shown (Figs 3 and 4). Both enzymes retaining lipid phosphatase activity, Ptenb WT (Fig 3) and Ptenb Y138L (Fig 4), were able to significantly suppress the hyperbranching vasculature phenotype at 3dpf (Figs 3–5). Micro- injection of the other Ptenb mutants (Fig 4) that lack Pten lipid phosphatase activity, Ptenb C124S and Ptenb G129E, did not suppress excessive sprouting of the intersegmental vessels. Also, co-injection of Ptenb G129E and Ptenb Y138L each at half the normal dose did not significantly rescue the hyperbranching phenotype. This might be due to the fact that only half the amount of lipid phoshphatase active Ptenb Y138L was injected compared to single injection of Ptenb Y138L. Therefore, we co-injected Ptenb G129E and Ptenb Y138L each at the normal dose. This, however, did not rescue the hyperbranching phenotype (S1 Fig), suggesting that Ptenb G129E might have a dominant-negative effect over Ptenb Y138L or that both phosphatase activities have to be present in the same molecule (see discussion).

From the z-stack images, we assessed the number of embryos showing hyperbranching of the intersegmental vessels at 3dpf and calculated the percentage of the observed phenotype according to the total number of embryos in each experimental condition (Fig 5). Again, as expected, the occurrence of the hyperbranching phenotype in the non-injected control is about 25% and can be significantly reduced by micro-injection of Ptenb-mCherry WT, to about 5%. From the quantification of hyperbranching, it appears that there might be a slight difference between the rescue capacity of Ptenb WT (5% phenotype observed) and Ptenb Y138L (8% phenotype observed). However, comparing the two conditions directly to one another and performing two-tailed Fisher’s exact test revealed that this difference is not significant. Neither are the differences between Ptenb G129E (20% phenotype observed) and Ptenb G129E/Ptenb Y138L injection (27% phenotype observed), which further confirms the observation that co-microinjection of the two complementary phosphatase mutants did not restore a Ptenb wild-type-like rescue capacity.

### Wild type Pten and Pten Y138L restore physiological pAkt levels in *ptena-/-ptenb-/-* double homozygous zebrafish embryos

**Fig 6:**
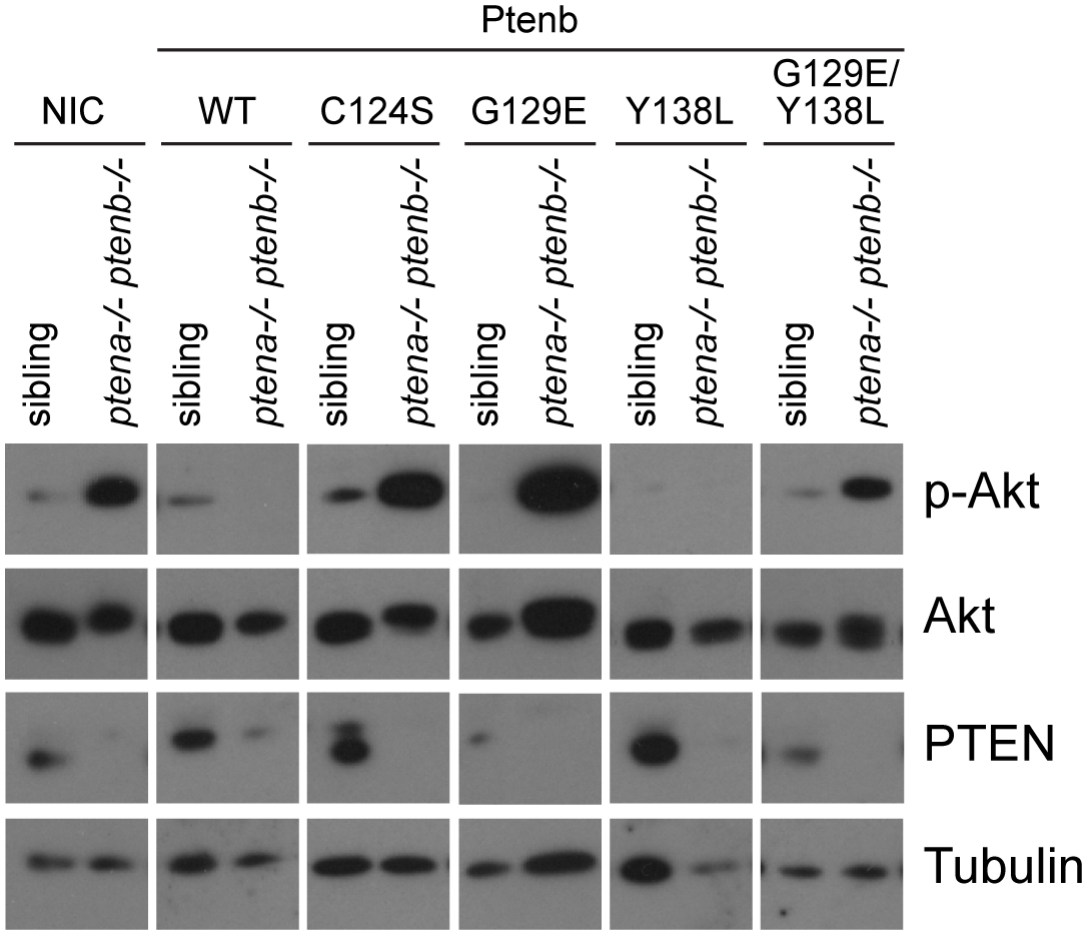
Only lipid phosphatase-active Pten restores normal pAkt levels at 4dpf. At 4dpf, single embryos from a *ptena+/-ptenb-/-* incross were cut in half. The trunk region was used for genotyping and the anterior half was lysed and processed for immunoblotting. Lysates from siblings and *ptena-/-ptenb-/-* embryos were run side by side on gels and blotted. The membranes were probed with phosphospecific anti-pAkt antibody (directed against pSer473), and subsequently sequentially stripped and probed with Akt-specific antibody, Tubulin-specific antibody as a loading control and PTEN-specific antibody, which only detects endogenous Ptena. The elevated pAkt levels in *pten* double homozygous embryos (see non-injected control) could be reduced to physiological levels by microinjection of either Ptenb WT or Ptenb Y138L RNA, indicating that both constructs have similar lipid phosphatase activities.

To assess the ability of the different Ptenb phosphatase mutants to antagonize PI3K/Akt-signaling *in vivo*, the embryos were genotyped at 4dpf, and the phosphorylation of downstream Akt in each embryo was detected by immunoblotting (Fig 6). These data further confirmed that both lipid phosphatase active Ptenb, Ptenb WT and Ptenb Y138L, but none of the other phosphatase mutants were capable of decreasing pAkt levels *in vivo*. We conclude that there may be a causal link between the characteristic hyperbranching of intersegmental vessels at 3dpf in *ptena*-/-*ptenb-/-* embryos and elevated PIP_3_/pAkt signaling, which is consistent with the notion that that Pten lipid phosphatase activity, but not protein phosphatase activity is required to suppress the *ptena-/-ptenb-/-* hyperbranching phenotype.

## Discussion

So far, the contribution of the distinct phosphatase activities of PTEN to its cellular functions has been mainly studied *in vitro* [14, 20–25, 28, 29, 31] and the few *in vivo* approaches did not include the PTEN protein phosphatase deficient mutant PTEN Y138L [26, 30, 50]. In order to address whether Pten lipid and protein phosphatase activity are required for correct embryonic development, we investigated the capacity of Pten mutants to functionally rescue the pleiotropic phenotype of *ptena-/-ptenb-/-* embryos. We found that Pten lipid and protein phosphatase are both required for normal embryonic development at 4 dpf and that lipid phosphatase activity is required for normal angiogenesis at 3 dpf. Consistent with our results at 4dpf, *Tibarewal et al*. reported that in Matrigel in *in vitro* assays with UM87G cells [29], only re-expression of wild type PTEN but of none of the PTEN phosphatase mutants, not even co-expression of PTEN G129E and PTEN Y138L, suppressed cell invasion in spite of the fact that PTEN Y138L restored normal pAkt levels. Based on these *in vitro* results and the finding that the main protein substrate of PTEN could be its own C-terminal tail, more specifically Thr366, *Tibarewal et al*. speculated that both phosphatase activities might have to be present on the same PTEN molecule in order to fulfill certain biological functions. In fact, they demonstrated that PTEN Y138L is hyperphosphorylated on Thr366, caused by its lack of protein phosphatase activity, and that mutation of this residue to alanine, T366A, restored the capacity of PTEN Y138L to suppress cell invasion [29]. Anyhow, we realized that in our experiments the mCherry signal was already quite difficult to detect at 4dpf, suggesting that at these relatively late developmental stages exogenous mRNA might have been degraded and hence the rescuing effect of Pten might be diminished. The limited lifespan of mRNA molecules *in vivo* is a common drawback of the microinjection technique and is the reason why achieving a long-lasting and complete rescue by microinjection of synthetic mRNA is virtually impossible.

To avoid the possible effects of RNA instability on the rescue capacity of Pten at later stages in development, we focused on an aspect of the *ptena-/-ptenb-/-* phenotype that emerges earlier during embryonic development: the characteristic hyperbranching of the intersegmental vessels at 3dpf [43]. Angiogenesis, the sprouting of new blood vessels from existing vasculature, is a highly regulated process during zebrafish embryonic development that requires a fine-tuned interplay between different signaling pathways, including Vegf signaling (vascular endothelial growth factor; artery and lymph vessel formation) and Bmp signaling (bone morphogenetic proteins; vein formation), as well as secondarily involved pathways, including for example Notch signaling, Semaphorin-Plexin signaling and the PI3K/Akt(PKB)/PTEN-axis. Upon binding of Vegfa to VEGFR2, the Vegfa receptor, PI3K signaling gets activated (as well as phospholipase Cγ1 and Src family kinases), promoting proliferation and differentiation of vascular endothelial cells and thereby inducing angiogenensis [51–53]. The work of various groups previously unveiled that PTEN, the main antagonist of PI3K signaling, suppresses this pathway *in vitro* [54] and lack of PTEN has been associated with enhanced angiogenesis in gastric cancer patients [55]. We reported that *pten* double homozygous zebrafish develop an ectopic vascular hyperbranching phenotype [43] and that this phenotype is rescued either by re-introducing Pten mRNA at the one-cell stage or by treating embryos with LY294002, a specific PI3K inhibitor, thereby proving that Pten suppresses angiogenesis *in vivo* mainly via decreasing phosphorylated Akt levels.

We were intrigued to find out whether the lipid phosphatase activity of Pten is sufficient to rescue the hyperbranching vessel phenotype or whether Pten requires both phosphatase activities in order to suppress angiogenensis. Our results revealed that wild type Ptenb and Ptenb Y138L, both possessing lipid phosphatase activity, were the only Ptenb constructs that significantly prevented the development of hyperbranching trunk vasculature in the double homozygous *pten* embryos at 3dpf. This indicates that Pten lipid phosphatase activity is required to suppress angiogenesis *in vivo*.

Detection of pAkt levels at 4dpf by immunoblotting further confirmed that both Ptenb WT and Ptenb Y138L, are capable of restoring physiological pAkt levels at this developmental stage. Immunoblotting suggested that Ptenb Y138L was more active than Ptenb WT, since it also suppressed pAkt levels in the micro-injected siblings. It would be interesting to address this question in further studies. To our surprise, co-microinjection of Ptenb-eGFP G129E and Ptenb-mCherry Y138L did not significantly rescue the hyperbranching vessel phenotype at 3dpf, whereas microinjection of Ptenb-mCherry Y138L by itself did rescue. This may be due to a dominant negative effect of Ptenb G129E on Ptenb Y138L, for example by dimerization [26]. *In vivo* studies in mice revealed that both mutations of Pten that abolish lipid phosphatase activity, C124S and G129E, have a dominant negative effect over wild type Pten in a heterozygous setting (Pten^+/C124S^ or Pten^+/G129E^), rendering mice with an amplified tumor spectrum compared to mice affected by Pten loss of heterozygosity (Pten^+/-^), an effect that can be explained by PTEN heterodimerization [26]. However, another explanation for the observed lack of a full rescue upon co-expression is that both phosphatase activities may need to be present in the same Pten molecule. Complete PTEN phosphatase functionality seems to be especially important to control complex biological processes such as invasion [29] and embryonic development.

We further believe that, as proposed by *Tibarewal et al*., certain biological functions of PTEN could either depend on lipid phosphatase-independent mechanisms, exerted by protein-protein interactions, or on lipid phosphatase activity of PTEN towards a specific spatial PIP_3_ pool that does not correlate with pAkt levels, as for example membrane ruffling and cell polarity [29]. This hypothesis would also explain why the Y138C mutation of PTEN has been positively selected for in the metastatic small cell lung cancer cell line NCI-H196 [29, 34, 41, 56, 57]. *Lyu et al*. recently reported that PTEN protein phosphatase activity but not lipid phosphatase activity is essential for the regulation of neuronal progenitor cell differentiation and that PTEN likely exerts its protein phosphatase-dependent function by dephosphorylating CREB [31]. Ultimately, cell motility, invasion, cell polarity and differentiation are all crucial processes that have to be highly regulated for correct embryonic development and therefore it is not surprising that some features of the *pten* double homozygous phenotype are controlled by PI3K/Akt-independent PTEN functions. Our results indicate that the role of Pten in antagonizing angiogenesis signaling is crucially dependent on its ability to dephosphorylate PIP_3_, whereas its role in zebrafish embryonic development likely depends on additional functions of Pten that are independent from suppressing the PI3K/Akt-signaling axis.

## Supporting Information

S1 Fig Co-injection of Ptenb-mCherry G129E and Ptenb-mCherry Y138L did not rescue developmental defects in ptena-/-ptenb-/- zebrafish embryos. Zebrafish embryos from a Tg(*kdrl*:*eGFP) ptena+/-ptenb-/-* incross were microinjected at the one-cell stage with mRNA encoding Ptenb-mCherry G129E and Ptenb-mCherry Y138L (300 pg each). At 3dpf the embryos were analyzed for the hyperbranching vessel phenotype by confocal live imaging on a Leica TCS-SPE microscope (top left panels; anterior to the left, 20x objective, 2μm z-stacks). Subsequently, the embryos were genotyped. Pictures show the trunk region distal from the urogenital opening of representative embryos; non-injected control embryos (NIC) were included for reference. Quantification of the number of embryos showing the typical *ptena*-/-*ptenb*-/- hyperbranching intersegmental vessel phenotype at 3dpf. Two-tailed Fisher’s Exact test indicated no statistically significant difference between NIC and G129E/Y138L-injected embryos (top right panel). The morphology of NIC embryos and G129E/Y138L-injected embryos was assessed at 4 dpf by brightfield microscopy (bottom left panels). Pictures show representative, genotyped embryos. Non-injected control embryos (NIC) were included for reference. Quantification of the embryos showing the typical *ptena*-/-*ptenb*-/- pleiptropic phenotype at 4dpf (bottom right panel). Two-tailed Fisher’s Exact test indicated no statistically significant difference between NIC and G129E/Y138L-injected embryos.
(PDF)
