# Supplementary figures and images for: Differential Requirement for Pten Lipid and Protein Phosphatase Activity during Zebrafish Embryonic Development
Source: PLoS One. 2016 Feb 5;11(2):e0148508. doi: 10.1371/journal.pone.0148508 (PMC4743836; doi:10.1371/journal.pone.0148508)

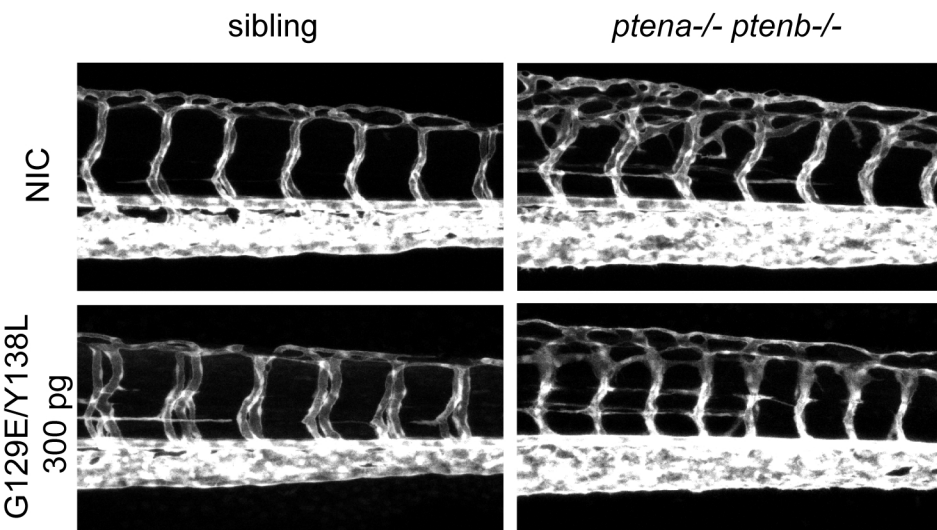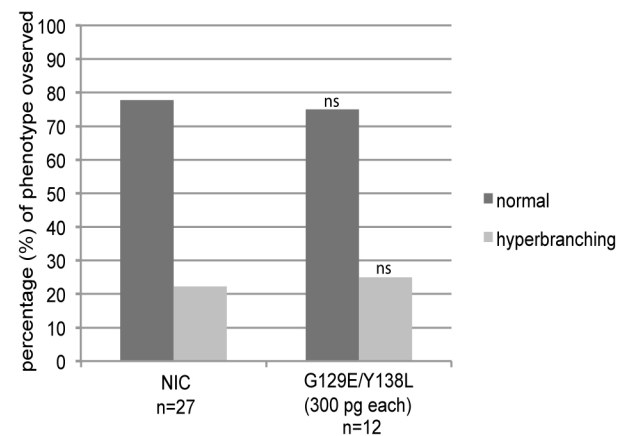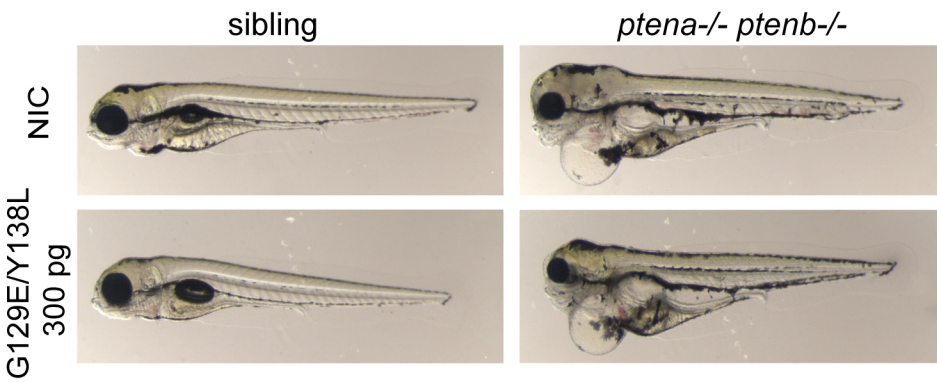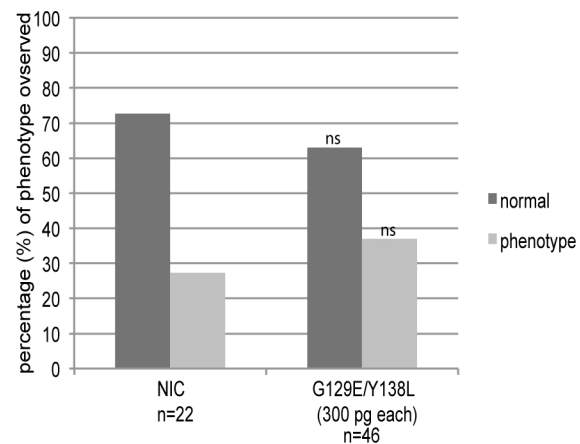

Supplement: S1 Fig — Zebrafish embryos from a Tg(kdrl:eGFP) ptena+/-ptenb-/- incross were microinjected at the one-cell stage with mRNA encoding Ptenb-mCherry G129E and Ptenb-mCherry Y138L (300 pg each). At 3dpf the embryos were analyzed for the hyperbranching vessel phenotype by confocal live imaging on a Leica TCS-SPE microscope (top left panels; anterior to the left, 20x objective, 2μm z-stacks). Subsequently, the embryos were genotyped. Pictures show the trunk region distal from the urogenital opening of representative embryos; non-injected control embryos (NIC) were included for reference. Quantification of the number of embryos showing the typical ptena-/-ptenb-/- hyperbranching intersegmental vessel phenotype at 3dpf. Two-tailed Fisher’s Exact test indicated no statistically significant difference between NIC and G129E/Y138L-injected embryos (top right panel). The morphology of NIC embryos and G129E/Y138L-injected embryos was assessed at 4 dpf by brightfield microscopy (bottom left panels). Pictures show representative, genotyped embryos. Non-injected control embryos (NIC) were included for reference. Quantification of the embryos showing the typical ptena-/-ptenb-/- pleiptropic phenotype at 4dpf (bottom right panel). Two-tailed Fisher’s Exact test indicated no statistically significant difference between NIC and G129E/Y138L-injected embryos. (PDF) [file pone.0148508.s001.pdf]
